# Synchronous Hepatocellular and Intrahepatic Cholangiocellular Carcinoma With Predominant Ductal Plate Malformation Pattern. A Case Report and Review of the Literature

**DOI:** 10.1002/cnr2.70085

**Published:** 2025-02-13

**Authors:** Rita Szodorai, Emőke Fülöp, Andrei Fülöp, Radu Mircea Neagoe, Simona Gurzu

**Affiliations:** ^1^ Department of Pathology George Emil Palade University of Medicine, Pharmacy, Science and Technology Târgu‐Mureș Romania; ^2^ Department of Pathology Clinical County Emergency Hospital Târgu‐Mureș Romania; ^3^ Department of Radiolology Clinical County Emergency Hospital Târgu‐Mureș Romania; ^4^ Department of Surgery George Emil Palade University of Medicine, Pharmacy, Science and Technology Târgu‐Mureș Romania; ^5^ Research Center for Oncology and Translational Medicine (CCOMT) Târgu‐Mureș Romania; ^6^ Romanian Academy of Medical Sciences Bucharest Romania

**Keywords:** case report, ductal plate malformation, hepatocellular carcinoma, synchronous tumors

## Abstract

**Background:**

Synchronous occurrence of hepatocellular carcinoma (HCC) and intrahepatic cholangiocarcinoma (ICC) is extremely rare.

**Case:**

A 67‐year‐old male was admitted to the hospital with hemoperitoneum caused by a liver mass rupture and elevated serum liver enzymes. Abdominal magnetic resonance imaging revealed a solid mass, with 38 mm in maximum diameter, located in the fifth/sixth segments of the liver, suggesting an HCC. Emergency surgery was performed and a second liver mass in the fourth segment was identified intraoperatively, with 20 mm in maximum diameter. Hepatic resection of the affected segments was performed with free resection margins. Histopathological examination revealed the synchronous occurrence of HCC and ICC with a predominant ductal plate malformation pattern. The patient is still alive at 41 months after its first surgery.

**Conclusions:**

In patients with HCC, a proper intraoperative assessment is indicated, in addition to imaging investigations, to detect synchronous lesions that can change the therapeutic approach. This is the first case ever reported in the literature in which synchronous HCC and ICC with a predominant ductal plate malformation pattern were incidentally diagnosed in a patient with hemoperitoneum.

## 1 Introduction

Liver cancer is the seventh most common cancer worldwide and the second most common cause of cancer mortality. GLOBOCAN 2018 estimated that by 2025, over 1 million individuals will be affected by liver cancer annually [1]. According to the World Health Organization (WHO), primary liver carcinomas can be histologically classified into three major types: hepatocellular carcinoma (HCC), intrahepatic cholangiocarcinoma (ICC), and combined hepatocellular cholangiocarcinoma (cHCC‐ICC). As HCC and ICC do not have the same origin, they usually develop independently of each other. Less than 0.8%–1% of cases are synchronous (sHCC‐ICC), and most of these were published as case reports [2, 3, 4, 5, 6].

We present the unusual association of HCC with a new subtype of ICC, known as “ductal plate malformation (DPM),” in a patient with hemoperitoneum. As no similar cases were reported in Medline or Web of Science databases, the clinicopathological features of this rare entity will be presented based on an extensive review of the literature.

## 2 Case

### 2.1 Clinical Findings

A 67‐year‐old male patient was admitted to the Emergency Department of Emergency County Clinical Hospital of Targu‐Mures, Romania, complaining of right hypochondrial pain, general weakness, melena, and weight loss.

### 2.2 Timeline

**FIGURE 1:**
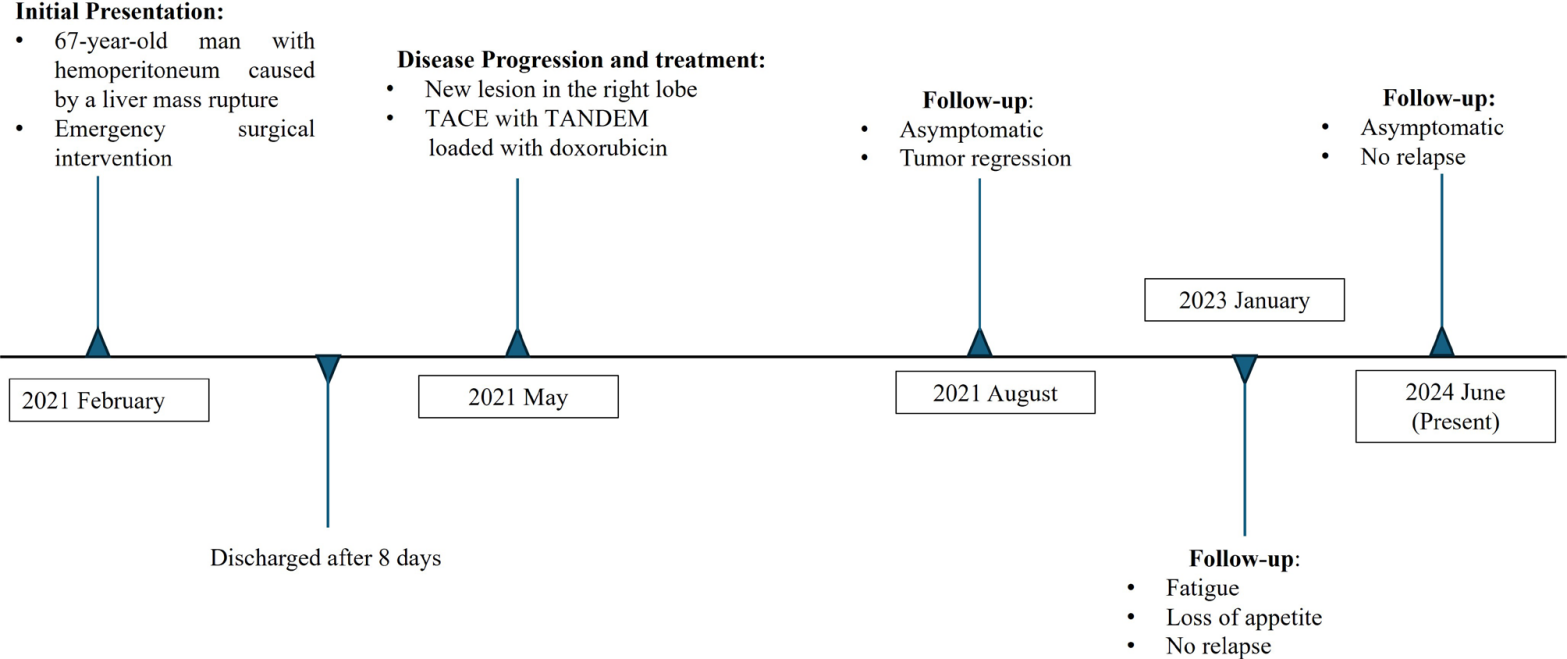
Timeline of a 67‐year‐old patient with synchronous HCC and ICC with a DPM component.

The 67‐year‐old male patient had previously been diagnosed with systemic hypertension and diabetes mellitus type 2. He was a smoker and had been abstinent from alcohol for 1 year. The patient's medical history was negative for drug consumption, blood transfusion, hepatitis, and liver cirrhosis, and there was no significant family history of liver disease. At the present admission, abdominal magnetic resonance imaging (MRI) revealed a hemoperitoneum that was caused by rupture of a liver tumor and emergent surgery was decided (Figure 1).

### 2.3 Diagnostic Assessment

**FIGURE 2:**
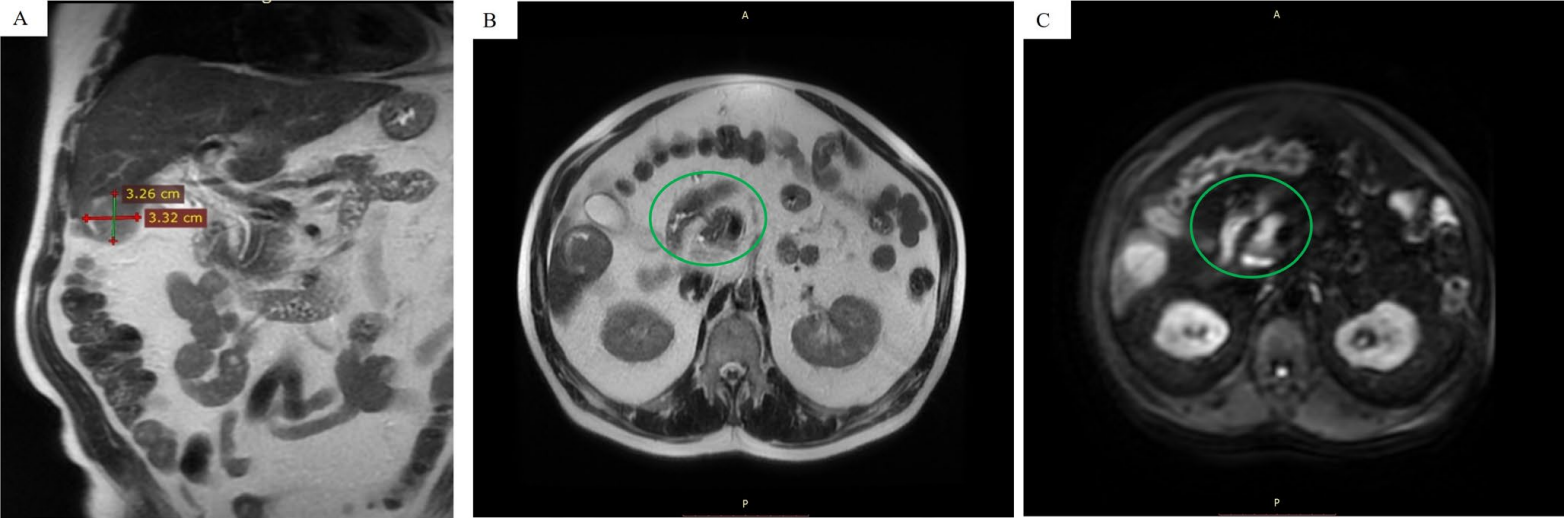
Abdominal magnetic resonance imaging (MRI) indicates a tumor mass located in the sixth segment of the liver—(A) Hyperintense round/oval nodule in the right lobe; (B) heterogeneity appearance of the HCC; and (C) DWI shows diffusion restriction of the nodule.

The MRI revealed hemoperitoneum and a relatively well‐defined tumor mass measuring 33 × 33 × 41 mm^3^ located in the fifth/sixth segments of the liver. The tumor, which was called “tumor A,” had low signal intensity on T1‐weighted imaging (T1WI) and high intensity on T2WI, with arterial‐enhancing and delayed wash‐out and diffusion restriction as indicators of malignancy, suggesting an HCC (Figure 2).

The laboratory findings indicated leukocytosis (11.79 × 10^3^ U/L, normal value: 3.6–10 × 10^3^ U/L) and anemia with low erythrocyte count (2.659 × 10^6^ U/L, normal value: 4.0–6.0 × 10^6^ U/L), hemoglobin (8.57 g/dL, normal value: 13–17 g/dL), and hematocrit (26.5%, normal value: 39.0%–54.0%). Low platelet count (128.7 × 10^3^/μL, normal value: 150.0–450.0 × 10^3^ U/L) and hypokalemia (potassium: 3.2 mmol/L, normal value: 3.5–5.1 mmol/L) were also reported. Mildly elevated values were recorded for liver enzymes, including aspartate aminotransferase (AST) (53 U/L, normal value: 5–34 U/L) and alanine aminotransferase (ALT) (178 U/L, normal value: 0–55 U/L) and for creatinine (3.3 mg/dL, normal value: 0.5–1.8 mg/dL).

### 2.4 Therapeutic Intervention

As the hemoperitoneum was believed to be caused by the tumor rupture and the patient showed uncorrectable coagulopathy, an emergency segmentectomy was performed. During surgery, another relatively well‐defined nodular structure was removed from the fourth segment of the liver and sent to the Pathology department for further examination. It was called “tumor B.”

As the patient showed a high serum level of creatinine (over 3 mg/dL) and uncorrectable coagulopathy, and the patient was treated during the COVID‐19 pandemic, based on the internal protocol of our hospital, it was not allowed to use contrasting substances before MRI. Consequently, we were unable to obtain imaging data that would reveal tumor B in the MRI scans, even in retrospective review. Additionally, it is noteworthy that the lesion does not exhibit diffusion restriction, and the tumor site was discernible in isosignal on T2‐weighted sequences. For this reason, tumor B was identified during surgery only.

### 2.5 Pathology Findings

**FIGURE 3:**
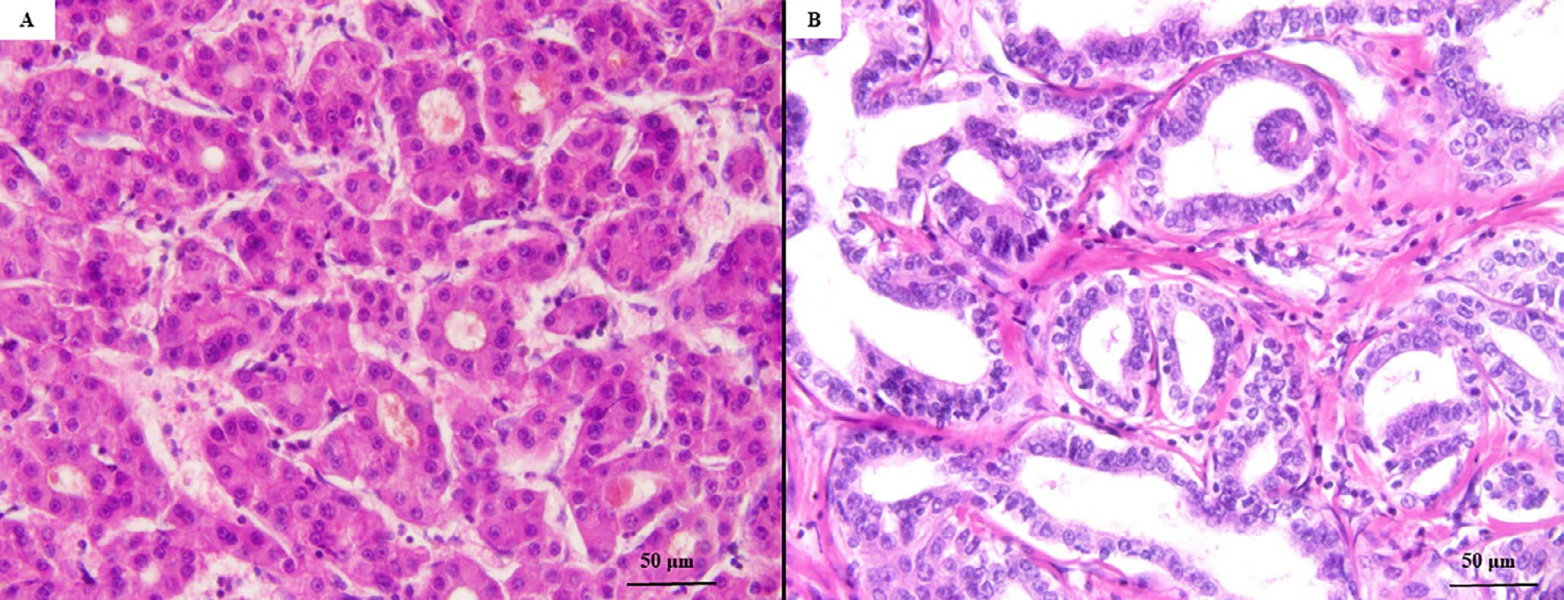
The histopathological features of the two tumors of the liver, emphasized with hematoxylin and eosin staining. The tumor developed in S5–S6 of the liver—Moderately differentiated hepatocellular carcinoma (A, ×40). The S4 resected tumor—Well‐differentiated intrahepatic cholangiocarcinoma DPM type (B, ×40).

**FIGURE 4:**
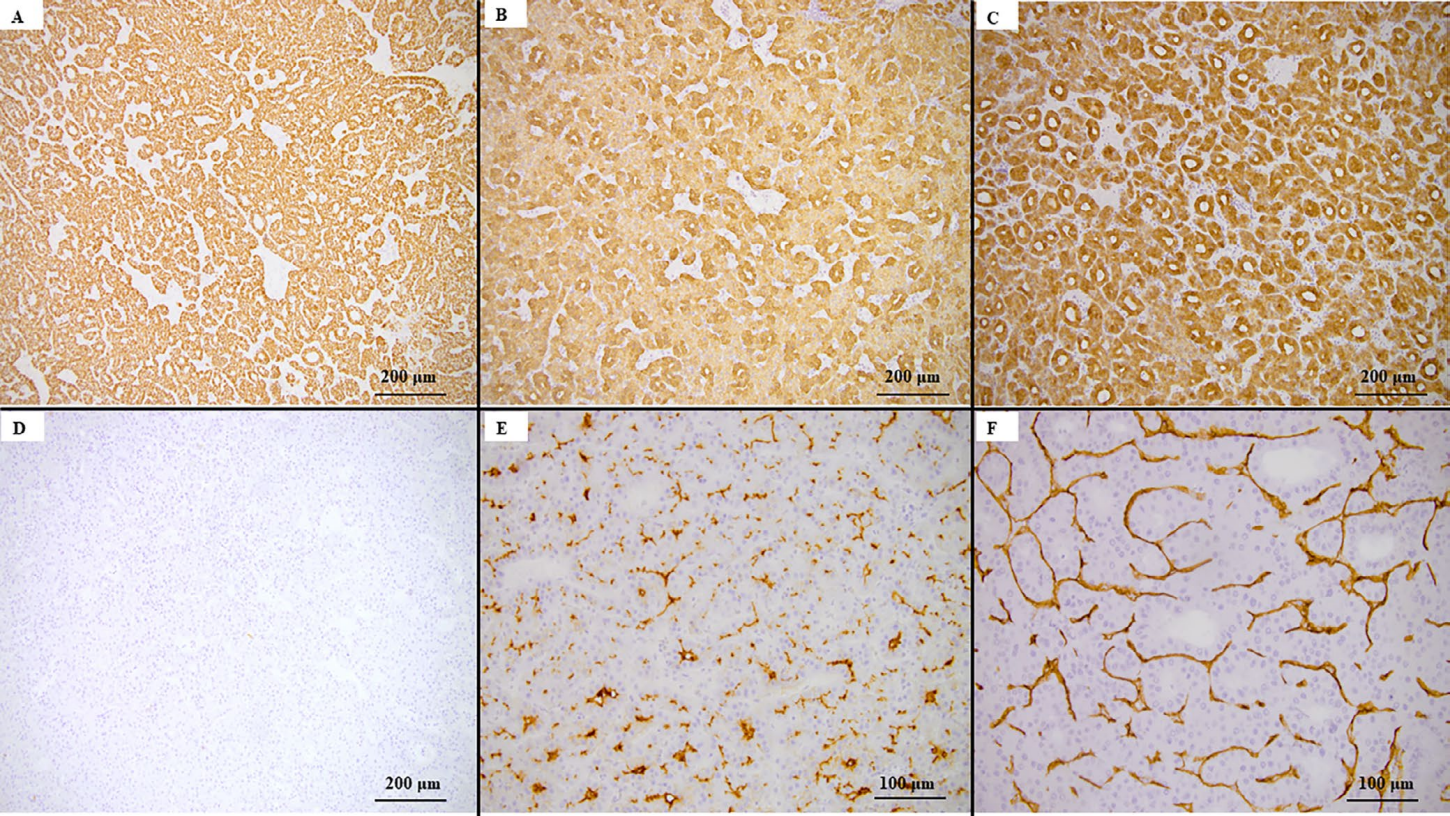
Immunohistochemical profile of HCC. The tumor cells reveal positivity for HepPar1 (A, ×10), cytokeratin 8 (B, ×10), and cytokeratin 18 (C, ×10), without expression for cytokeratin 7 (D, ×10); CD10 (E, ×20) and CD34 (F, ×20) are expressed in pericanalicular/perisinusoidal spaces.

The liver mass excised from the fifth/sixth segments, which was emphasized on MRI examination as “tumor A” proved to be a well‐circumscribed solid tumor of 38 mm in maximum diameter with micronodular aspects on the cut section and free resection margins. Under microscope, the tumor consisted of proliferating tubular structures with trabecular and pseudoglandular patterns. The tumor cells were polygonal in shape and exhibited nuclear atypia, with a high nucleocytoplasmic ratio, irregular nuclear membrane, multinucleation, and prominent nucleoli (Figure 3). Neither lymphovascular nor perineural invasion was identified. The satellitosis phenomenon was not detected, and the peri‐tumoral parenchyma showed pseudo‐nodules that were specific to cirrhosis. Immunohistochemical (IHC) analysis revealed diffuse cytoplasmatic expression of HepPar1 and cytokeratins 8 (CTK8) and 18 (CTK18). Canalicular (CD10+) and sinusoidal (CD34+) staining patterns were seen. No positivity for CTK7 and carcinoembryonic antigen (CEA) was observed. The histological and IHC patterns suggested the diagnosis of a moderately differentiated (G2) HCC (Figure 4).

**FIGURE 5:**
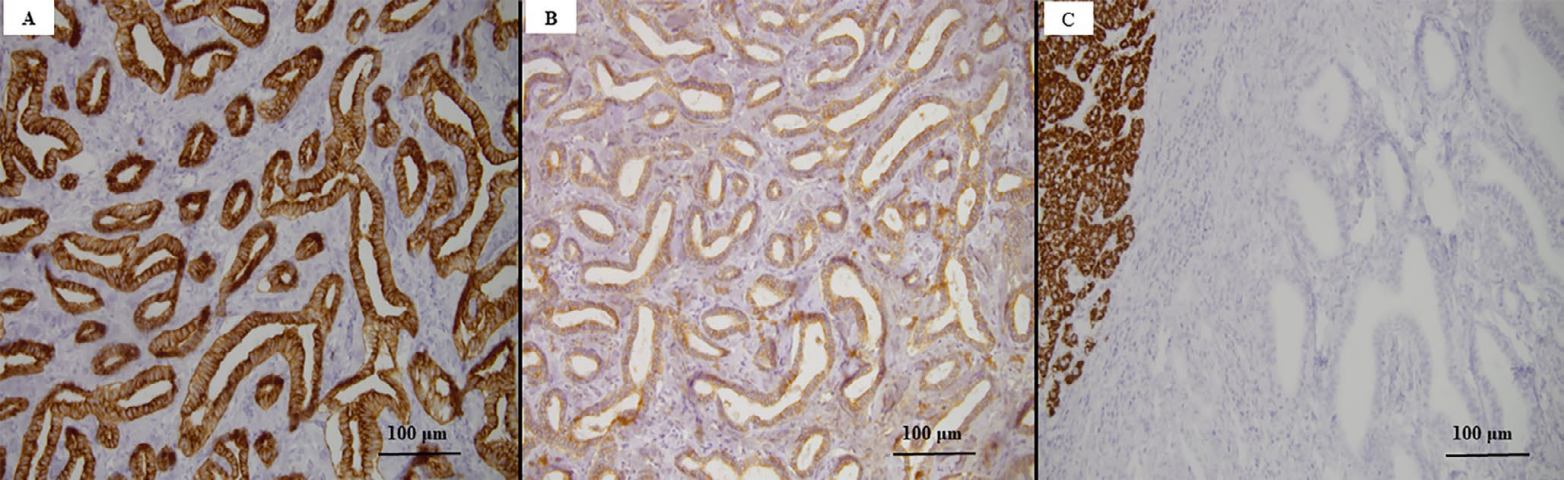
Immunohistochemical profile of ICC‐DPM type reveals positivity for cytokeratin 7 (A, ×20) and cytokeratin 19 (B, ×20), without expression for HepPar1 (C, ×20).

Tumor B involved the fourth segment of the liver, which was solid and 20 mm in diameter. On the cut section, the whitish tumor had poor boundaries. Microscopically, the tumor parenchyma consisted of irregular, large, tortuous glandular structures surrounded by a fibrous stroma. The irregularly dilated lumens were lined by a single layer of cuboidal or columnar eosinophilic cells and presented a high nucleocytoplasmic ratio. These features indicated the diagnosis of ICC with a DPM pattern. The nuclei were vesicular, with fine chromatin and small nucleoli, scarce mitoses, a Ki67 index of 10%, and lymphatic invasion (Figure 3). The adjacent liver parenchyma was steatotic, without any signs of cirrhosis. The tumor cells were marked by epithelial membrane antigen (EMA), CTK7, and CTK19, and did not express HepPar1 (Figure 5). The histological and IHC patterns confirmed the diagnosis of a well‐differentiated (G1) ICC with predominant DPM (ICC‐DPM type).

**FIGURE 6:**
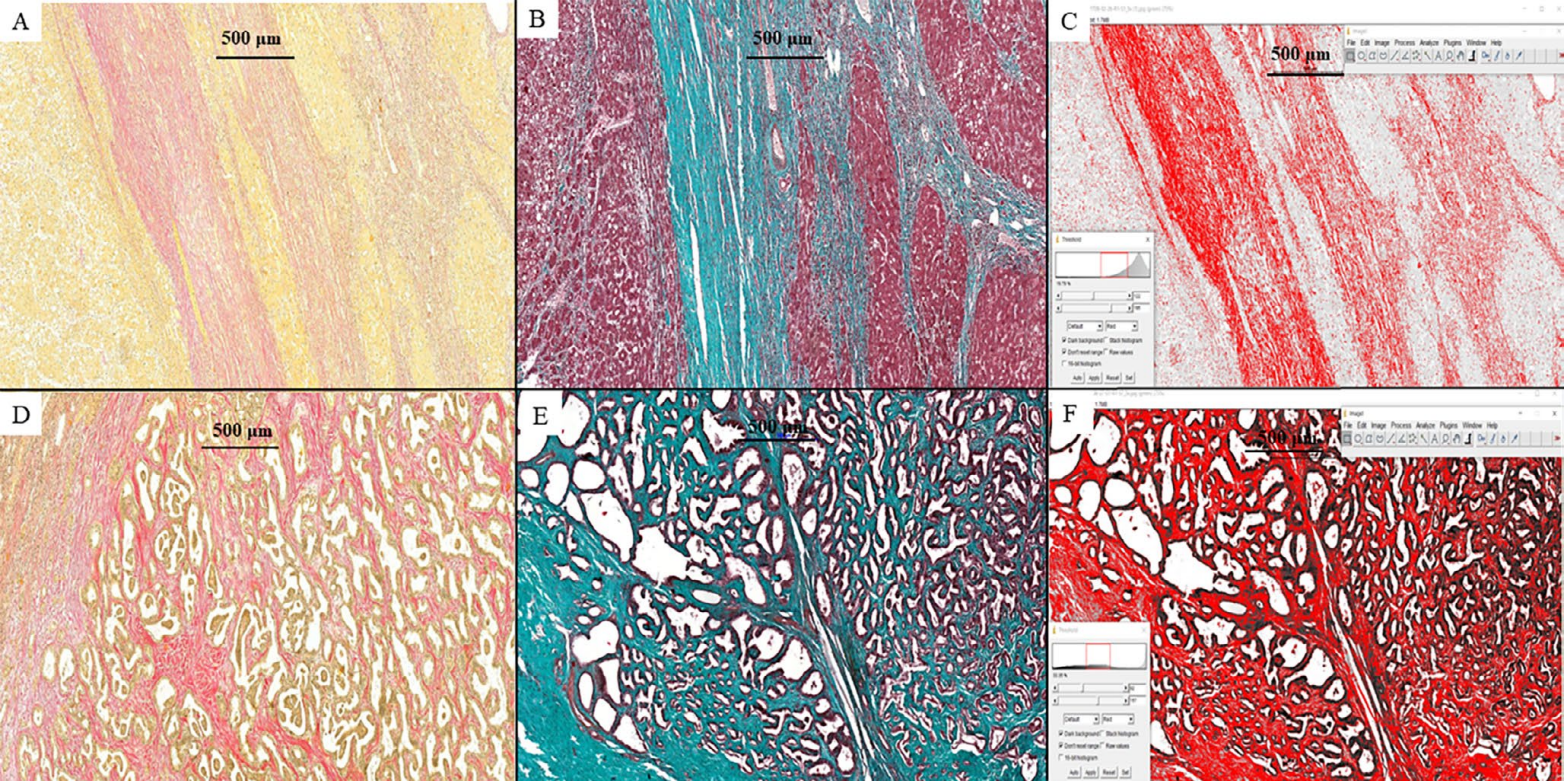
Peritumoral fibrosis, in HCC (A–C) and DPM component (D–F), revealed by Elastic‐Van Gieson (A and D, ×5) and Masson's trichrome stains (B and E, ×5), and quantified with the ImageJ software—the Threshold option (C&F, x5).

The amount of peritumoral fibrosis was highlighted with Elastic‐Van Gieson and Masson's trichrome stains (Figure 6). The percentage of area with fibrosis in the peritumoral region was estimated using the Threshold option in the ImageJ—image processing program. In HCC component, peritumoral fibrosis measured 19.79% of the background liver. Due to the ill‐defined feature of the DPM component, peritumoral fibrosis was analyzed in multiple sections, measuring 10.47%. Subsequently, the quantification of the abundant fibrotic stroma resulted in a measurement of 33.34%.

### 2.6 Follow‐Up and Outcomes

The postoperative evolution was uneventful, and the patient was discharged after 8 days. After 4 months, a new lesion was detected in the right lobe of the liver. Transcatheter arterial chemoembolization (TACE) with TANDEM loaded with doxorubicin was performed [7]. The patient is still alive 41 months after the first surgery (Figure 1).

## 3 Discussion

HCC and ICC, two histological forms of primary cancer of the liver, can occur independently of each other or can be identified as a cHCC‐ICC or sHCC‐ICC. These combined variants are known to be associated with shorter survival compared to classic HCC [5, 8, 9]. Moreover, HCC is hypothesized to exhibit tumor plasticity and the capacity to transform into ICC or vice versa [10]. In our case, we did not identify any areas that were suggestive of such a transformation. Histological signs of cirrhosis were only observed in the liver parenchyma surrounding the HCC and not the ICC. The obvious differences regarding the amount of peritumoral fibrosis were proved after using Elastic‐Van Gieson and Masson's trichrome stains. In the peritumoral area, fibrosis was expressed two times more in the HCC than in the ICC.

Although infection with hepatitis viruses (HCV) is known to predispose to cirrhosis and HCC, molecular modifications induced by HCV have also been described in double primary tumors [11, 12].

Synchronous occurrence of HCC and ICC‐DPM, previously unreported in the literature, makes the preoperative diagnosis and oncological decisions challenging. In over 20% of cases, imaging investigations are suggestive of HCC, independently of the histological variants of primary liver cancers [3, 5]. As HCC is mostly a multifocal tumor, multiple liver tumors are initially diagnosed as HCC, like our case, when the secondary tumor was identified intraoperatively only; pathological confirmation of the surgical specimens is necessary in such cases [3].

The clinical manifestations of synchronous HCC‐ICC are not specific. High serum levels of alpha‐fetoprotein (AFP) and CA19‐9 are detected in 29% of synchronous HCC‐ICC compared with only 6% of pure ICCs and 9% of pure HCCs [10, 13, 14, 15]. These markers are neither sensitive nor specific.

Imaging investigations can be useful for diagnosis. Although not helpful in all cases, computed tomography (CT) or MRI can help to distinguish the two histological variants, only if contrasting substances are administered. ICC is characterized by “delayed reinforcement,” which is a peripherally enhanced area that occurs during the early phase and is followed by mild centripetal progression of enhancement over time on both dynamic CT and MRI. In contrast, the main feature of HCC is “fast wash‐in and fast wash‐out” [10, 16, 17, 18]. Therefore, even if the prevalence of this type of liver cancer is very low, sHCC‐ICC should be suspected in patients with a simultaneous increase in serum levels of AFP and CA19‐9 [10].

From a histopathological point of view, HCC is usually a well‐circumscribed, firm mass that appears from tan, yellow to green. Hemorrhage and necrosis are common on cut surfaces. ICC is usually a larger, nonencapsulated, white‐tan, or gray nodular mass with firm consistency [19]. Under microscope, HCC is a well‐vascularized tumor with a trabecular, pseudoglandular, or acinar pattern, possible bile or fibrin content, and specific loss of the reticulin network [19, 20, 21]. Most cases of HCC develop in patients with cirrhosis. Tumor cells are positive for IHC markers, such as HepPar1, arginase1, glypican 3, pCEA, and CD10, and negative for AE1/AE3, CK7, CK13, CK19, CK20, CDX2, and CEA. In contrast, ICC has desmoplastic stroma and resembles adenocarcinoma, with small glandular structures and frequent perineural invasion, which makes it difficult to differentiate from a pancreatic ductal adenocarcinoma metastasis. The commonly positive IHC markers of ICC are CK7, CK19, and EpCAM, and tumor cells are usually negative for HCC markers, such as HepPar1, arginase1, pCEA, CD10, AFP, and glypican 3 [22].

In addition to the synchronicity, in our case, the ICC component indicated a rare histological variant. The ICC‐DPM type is even more rarely encountered than combined HCC‐ICC. As most of the existing data are provided in case reports, the pathogenesis of the ICC‐DPM type remains unclear. Up to 2004, ICC‐DPM was considered a variant of combined HCC‐ICC; however, it was later reclassified by the WHO and recognized as a separate entity [4, 5].

DPM represents the persistence of the embryonic ductal plate after birth and appears in association with congenital hepatic fibrosis, polycystic liver, and kidney diseases, congenital biliary atresia, von Meyenburg complex, Caroli's disease, and acquired hepatobiliary diseases [23, 24].

ICC with DPM pattern was defined in 2012 by Nakanuma et al. as single tumors of the liver located predominantly in the right lobe. On the cut surface, they appear as a solid, whitish, or gray nodule with a relatively clear border, elastic‐firm consistency, and no capsule. The central part of the nodule might be hyalinous. Histologically, it consists of proliferation of irregular, elongated, tortuous glandular structures with dilated lumens lined with a single layer of ductule‐like patterns composed of cuboidal or low‐columnar carcinoma cells, with clear and scanty cytoplasm in a fibrous stroma. Tumor cells have vesicular nuclei with fine chromatin and small nucleoli. Mitoses are rare and atypical. These tumors are well to moderately differentiated, without microvascular or perineural invasion, as in our case. IHC diagnosis is based on positivity for EMA, EpCAM, NCAM, and CK19 [23].

Like other primary tumors of the liver, treatment of ICC‐DPM consists of surgical resection or TACE [7]; however, TACE results are limited for ICCs, which have a fibrotic stroma and fewer vascular components than HCCs. In our patient, hepatic segmentectomy for the primary tumors was followed by TACE 4 months after surgery. Although TACE is recommended as the first‐line treatment upon the tumor rupture too, association of renal failure and uncorrectable coagulopathy contraindicated this intraoperative management [25]. An important issue in the patient's response to TACE or antiangiogenic therapy refers to the amount of peri‐ or intra‐tumoral fibrosis. As the newly formed vessels are mostly immature and their genesis is mediated by vascular endothelial growth factor (VEGF), patients with tumors developed on the background of cirrhosis might respond to anti‐VEGF medications. On the contrary, COX‐2 intensity was proved higher in patients with the fibrosis levels returned to background [26, 27].

The present case highlights the role of a transdisciplinary team in the management of rare cancers, such as synchronous HCC and ICC‐DPM, which are challenging tumors, from diagnosis to therapy. It highlights the necessity of using contrasting substances for MRI‐based evaluation of any patient with HCC, the attentive intraoperative exploration of the liver, even if the imagistic description indicates a single tumor, and the importance of individualized therapy, based on imagistic and histological parameters.

## Author Contributions

**Rita Szodorai:** conceptualization, writing – original draft, formal analysis, data curation. **Emőke Fülöp:** conceptualization, methodology, formal analysis, resources. **Andrei Fülöp:** investigation, validation, formal analysis, visualization. **Radu Mircea Neagoe:** investigation, formal analysis. **Simona Gurzu:** writing – review and editing, supervision, resources, funding acquisition, conceptualization.

## Ethics Statement

The patient was transferred for further follow‐up to a private hospital. He declared that he feels healthy and trusts the medical personnel.

## Consent

Signed informed consent to perform the intervention and use the collected data and images for scientific purposes was obtained from the patient before surgery.

## Conflicts of Interest

The authors declare no conflicts of interest.

## Data Availability

Data sharing is not applicable to this article as no data sets were generated or analyzed during the current study.
